# The clinical impact of an extra virgin olive oil enriched mediterranean diet on metabolic syndrome: Lights and shadows of a nutraceutical approach

**DOI:** 10.3389/fnut.2022.980429

**Published:** 2022-08-04

**Authors:** Aurelio Seidita, Maurizio Soresi, Lydia Giannitrapani, Vita Di Stefano, Roberto Citarrella, Luigi Mirarchi, Antonella Cusimano, Giuseppa Augello, Antonio Carroccio, Juan Lucio Iovanna, Melchiorre Cervello

**Affiliations:** ^1^Unit of Internal Medicine, Department of Health Promotion Sciences, Maternal and Infant Care, Internal Medicine and Medical Specialties (PROMISE), University of Palermo, Palermo, Italy; ^2^Institute for Biomedical Research and Innovation (IRIB), National Research Council, Palermo, Italy; ^3^Department of Biological, Chemical and Pharmaceutical Sciences and Technologies (STEBICEF), University of Palermo, Palermo, Italy; ^4^Unit of Internal Medicine, “V. Cervello” Hospital, Ospedali Riuniti “Villa Sofia-Cervello”, Palermo, Italy; ^5^Department of Health Promotion Sciences, Maternal and Infant Care, Internal Medicine and Medical Specialties (PROMISE), University of Palermo, Palermo, Italy; ^6^Cancer Research Center of Marseille, Aix-Marseille University, CNRS, INSERM, Institut Paoli-Calmettes, CRCM, Marseille, France

**Keywords:** extra virgin olive oil (EVOO), nutraceuticals, functional foods, metabolic syndrome, cardiovascular disease, insulin resistance

## Abstract

For years it has been established that the only truly effective treatment of metabolic syndrome (MS) is lifestyle modification to prevent its cardiovascular (e.g., coronary artery disease and atherosclerosis), metabolic (e.g., diabetes mellitus), and hepatic (e.g., steatosis and non-alcoholic steatohepatitis) complications. The focal points of this approach are to increase physical activity and intake of a diet characterized by high quantities of fruits, vegetables, grains, fish, and low-fat dairy products, the so called mediterranean diet (MD); however, the added value of MD is the presence of extra virgin olive oil (EVOO), a healthy food with a high content of monounsaturated fatty acids, especially oleic acid, and variable concentrations (range 50–800 mg/kg) of phenols (oleuropein, ligstroside, and oleocanthal, and their derivatives, phenolic alcohols, such as hydroxytyrosol and tyrosol). Phenolic compounds not only determine EVOO’s main organoleptic qualities (oxidative stability, specific flavor, and taste features) but, theoretically, make it a source of antioxidant, anti-inflammatory, insulin-sensitizing, cardioprotective, antiatherogenic, neuroprotective, immunomodulatory, and anticancer activity. Although many studies have been carried out on EVOO’s clinical effects and attention toward this dietary approach (healthy and palatable food with strong nutraceutical activity) has become increasingly pressing, there are still many dark sides to be clarified, both in terms of actual clinical efficacy and biochemical and molecular activity. Thus, we reviewed the international literature, trying to show the state of the art about EVOO’s clinical properties to treat MS (along with correlated complications) and the future prospective of its nutraceutical use.

## Introduction

Metabolic syndrome (MS) is an increasingly pressing global health problem, affecting about 31% of the world’s population but predicted to increase over 50% in the next 15 years (1, 2). The National Cholesterol Education Program (NCEP) Adult Treatment Panel III (ATP III) (3) indicates MS when at least 3 of 5 conditions coexist: abdominal obesity, high triglyceride values, low high-density lipoprotein (HDL) values, high blood pressure, and impaired fasting glucose. These, both individually and in the context of MS, are known risk factors for metabolic and cardiovascular diseases (CVD) (4).

The only established, effective treatment of MS is lifestyle modification through increased physical activity, weight loss, and dietary intake high in fruits, vegetables, grains, fish, and low-fat dairy products: i.e., the mediterranean diet (MD) (4–6). Several studies have shown a direct correlation between MD adherence and overall reduction in mortality and morbidity (6–9).

An MD component believed to contribute a strong beneficial effect is extra virgin olive oil (EVOO), high in monounsaturated fatty acids (MUFAs) and with variable concentrations of phenols. These not only determine EVOO’s main organoleptic qualities (oxidative stability, specific flavor, and taste features) but make it a source of antioxidant, anti-inflammatory, insulin-sensitizing, cardioprotective, antiatherogenic, neuroprotective, and immunomodulatory activity (10). Although many studies have examined EVOO’s clinical effects and MD is seen as increasingly promising, there are still many uncertainties to be clarified regarding its clinical efficacy and biochemical activity.

We reviewed the international literature to summarize the state of the art about EVOO’s clinical properties for treating MS and the future prospects of its nutraceutical use.

## Extra virgin olive oil: What are we talking about?

Olive oil (OO) has an energetic function, transports fat-soluble vitamins, and makes foods more pleasant.

The composition of OO and EVOO is influenced by tree variety, agronomic conditions, production processes, period, harvesting method, and oil extraction system (11, 12).

Olive oil mostly consists of triglycerides (98–99%) and contains primarily MUFAs in the form of omega-9 oleic acid (C18:1); according to the International Olive Oil Council, its concentration must range from 55 to 83% of total fatty acids.

Olive oil also contains other MUFAs, such as omega-7 palmitoleic acid (C16:1), ranging from 0.3 to 3.5%, and traces of gadoleic/9-eicosenoic (C20:1 ω-11, 0.4%) and heptadecenoic acid (C17:1, 0.3%).

Extra virgin olive oil also contains polyunsaturated fatty acids (PUFAs) including linoleic acid (C18:2, ω-6) and α-linolenic acid (C18:3, ω-3), between 3 and 19% and 0.11 and 1.0%, respectively. EVOO’s lipid profile and high ω6/ω3 ratio have been linked to its protective effects on cardiovascular (CV), autoimmune and inflammatory disorders, but also its anti-thrombotic and blood pressure regulatory qualities, and ensuring oxidative stability for long shelf life (13–15).

Saturated fatty acids participate in the EVOO fatty acid profile: palmitic acid (C16:0, 7.8–17.3%), stearic acid (C18:0, 0.2–3.2%), arachidic acid (C20:0, 0.7%), margaric acid (C17:0, 0.3%), behenic acid (C22:0, 0.2%), lignoceric acid (C24:0, 0.2%), and myristic acid (C14:0, 0.03%).

The minor fraction of EVOO comprises substances responsible for its biological properties and sensory attributes (color, odor, flavor, taste, and aftertaste), primarily present in the mature drupe pulp and pits which are dissolved in the oil via natural or technological processes. Lower quantities of squalene (3–6 g/kg) and phytosterols (β-sitosterol, campesterol, and stigmasterol, in free and esterified forms) (0.8–2.6 g/kg) are present in EVOO. Finally, soluble vitamins (β-carotene and tocopherols), pigments (carotenes and chlorophyll), alcohol triterpene, and especially polyphenols are present in minor quantities.

Phenolic compounds include about 30 molecules from different chemical classes: phenolic alcohols, such as hydroxytyrosol (HT) and tyrosol (Tyr), phenolic acids, flavones, lignans, and secoiridoids. The latter group represents the largest fraction. The principal ones are the aglycone forms of oleuropein and ligstroside, the dialdehydic forms of their decarboxymethylated derivatives, known as oleacein and oleocanthal (Supplementary Table 1). In OO, the content of phenolic compounds ranges from 50 to 1000 mg/kg. The secoiridoids act as natural antioxidants protecting EVOO against autoxidation during storage and are responsible for its bitter and pungent qualities. Much evidence indicates that EVOO’s phenolic compounds can exert biological activities due to their antioxidant, anti-inflammatory, and chemo-preventive properties (16, 17).

In humans, Tyr and HT intestinal absorption occurs in a dose-dependent way with a percentage ranging from 40 to 95% and is strictly dependent on the polarity of their chemical structure (18, 19). Part of these polyphenols, in particular aglycone secoiridoids, can be hydrolyzed at the gastric level, with a time-dependent process, transforming into free Tyr and HT (20); the glycosylated forms do not suffer hydrolysis processes and, together with other polyphenols, pass through the small intestine where they are absorbed by enterocytes via a bidirectional passive diffusion mechanism (19). Once absorbed, polyphenols undergo phase II transformation metabolism, which substantially reduces their bioavailability. The most represented metabolites in plasma are the O-glucuronidated forms of Tyr and HT (21) and, to a lesser extent, homovanillic acid, homovanillic acid sulfate, and HT acetate sulfate (22). Both the unmodified forms and the metabolites of the polyphenol subclasses are ubiquitously distributed in the organism, depositing, in a concentration-dependent way, in certain organs, such as the brain, liver, and kidneys (23).

The clearance of polyphenols and their metabolites essentially occurs via kidney excretion (17, 24).

Based on health studies, in 2011 the European Food Safety Authority (EFSA) authorized a functional health claim on EVOO polyphenols that they “contribute to the protection of blood lipids from oxidative stress.” This benefit emerges with a minimum concentration of 5 mg of HT and its derivatives in 20 g of EVOO (25). Nevertheless, this is a contested point. Originally, Regulation (EC) No 1924/2006 included several health claims for OO polyphenols [for details see European Community, (26)]. In 2011, EFSA was asked about these claims and concluded: “that a cause and effect relationship has been established between the consumption of OO polyphenols and protection of low-density lipoproteins (LDL) particles from oxidative damage” (25). All others health claims were excluded, primarily for inadequate evidence from human studies, because they were “generic and not specific,” or because they did not comply with the criteria in Reg. 1924/2006 (27). In 2012, the European Council updated the regulation to implement this opinion (28). Since then, several studies have analyzed other health claims, reporting new data and raising more issues, including the lack of consistent studies correlating the chemical features and human benefits, especially anti-inflammatory properties of OO phenolic compounds (27).

**FIGURE 1:**
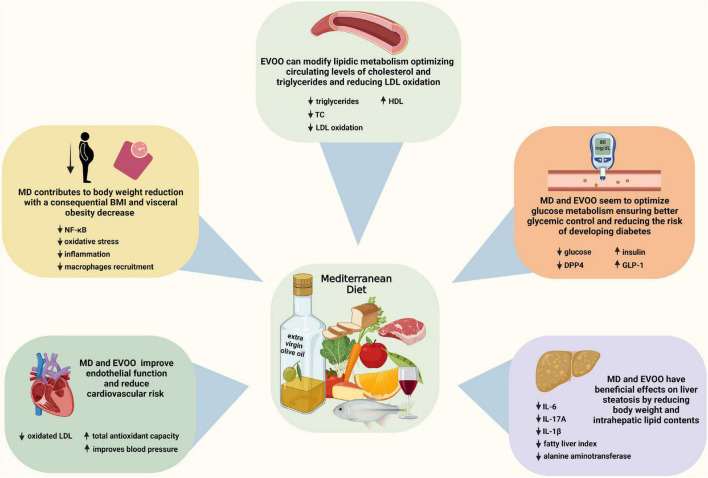
Beneficial effects of the mediterranean diet (MD) and extra virgin olive oil. Some key alterations implicated in MD and EVOO effects are presented. Created with BioRender.com.

An overview of some of the beneficial effects of MD and EVOO is presented in Figure 1.

## Extra virgin olive oil-induced metabolic pathways

Multiple conditions affect EVOO’s biochemical effects: component composition and concentration, absorption, and metabolism. Exposing EVOO to high temperatures or long cooking times might substantially alter its polyphenol content (29). A substantial role could be attributed to the intestinal microbiota, which can modify the metabolism of EVOO’s components and alter their absorption (30).

Several researchers focused on EVOO polyphenols’ reduction of oxidative stress. Some studies demonstrated that polyphenols bind LDLs and prevent their oxidation by free radicals (31, 32). Although the molecular mechanism underlying EVOO’s antioxidant activity is not fully defined, some authors assumed that it could modulate the expression of nuclear factor (erythroid-derived 2)-like 2 (Nrf2), consequently increasing antioxidant molecule expression (33, 34). This activity would explain the increase in glutathione turnover after a high EVOO content meal, with increased glutathione peroxidase and glutathione reductase activity and the reduction of post-prandial blood levels of lipid peroxide, protein carbonyl, and plasma hydrogen peroxide, probably because of NADPH oxidase activity decrease (34–37).

Moreover, HT and oleocanthal seem to inhibit copper-induced LDL oxidation by chelating metals and scavenging free radicals (38).

Extra virgin olive oil, due to its high MUFA content, significantly reduces concentrations of total cholesterol (TC) and LDL-cholesterol (LDL-C), decreasing TC/HDL and LDL/HDL ratios (6, 39). In this context, polyphenols act synergistically with MUFAs, causing both an inhibition of pancreatic lipases, delaying the post-prandial lipemic peak (40), and rapid lipid clearance (41).

Extra virgin olive oil also exerts anti-inflammatory activity by modulating the activation of pro-inflammatory genes and reducing pro-inflammatory cytokine expression. Several studies proved a reduction in serum phlogosis markers, both immediately after EVOO rich meals and in long-term consumption (6).

Among the downregulated pro-inflammatory molecules are interleukin-6 (IL-6), visfatin, tumor necrosis factor-α (TNF-α), IL-1β, interferon-γ (IFN-γ), and cyclooxygenase-2 (COX-2) which have been analyzed in human (42) and animal (43, 44) models, while IL-1, IL-3, and IL-8 have been analyzed in human peripheral blood mononuclear cells only (45). Finally, TNF-α, IL-6, and IL-17 production was studied in the splenocytes of a mouse model of systemic lupus erythematosus (46). In addition, an increase of anti-inflammatory cytokine IL-10 levels (43) and inhibition of some cell adhesion molecules (VCAM-1 and ICAM-1) have been reported (47). COX-2, LRP1, and MCP-1 are some of the genes modulated in this anti-inflammatory activity. (48). In studies specifically focused on MS patients, EVOO significantly reduced C-reactive protein (CRP) values, IL-6, IL-7, and IL-18 plasma levels (49), and pro-inflammatory molecule gene expression (50).

Extra virgin olive oil-enriched MD intake for 4 weeks helps normal endothelial function by promoting post-prandial vasodilation in patients with hypercholesterolemia (51). Similar effects have been demonstrated in other populations in which a greater bioavailability of nitric oxide (NO) (52) and a reduction of its urinary catabolites were proven. Some authors indicate that chronic EVOO intake can increase endothelial progenitor cells (53). These effects plausibly depend on reducing oxidative stress and the greater stability of the EVOO-induced endothelial cell genome (54). Recent *in vitro* studies tested EVOO’s effects on endothelial function, showing that HT can increase NO synthesis (55) and that EVOO’s polyphenols can modulate NADPH oxidase activity, reducing vascular endothelial growth factor production and reducing cellular migration and reactive oxygen species genesis (56).

Extra virgin olive oil’s modulation of nuclear factor κB (NF-κB), a transcription factor regulating gene transcription in cytokines, chemokines, adhesion molecules, inflammatory proteins and COX-2 (and several others), plays a significant role. NF-κB is also involved in inflammatory processes related to atherogenesis. EVOO, specifically its phenols, might modulate NF-κB expression, reducing the inflammatory cascade (57).

While many studies agree on these beneficial effects, there are conflicting opinions on EVOO’s ability to modulate platelet aggregation and coagulation. Some studies seem to indicate that both MUFAs and polyphenols could reduce platelet aggregation, probably inhibiting thromboxane A2 synthesis (56, 58–60). EVOO’s MUFAs might reduce factor VII, tissue factor, and plasminogen activator inhibitor-1 procoagulant activity (61–63); however, the small number of studies with discordant results require further analysis to clarify EVOO’s effect on the coagulation cascade.

## Extra virgin olive oil’s effects on metabolic syndrome components

Since in the 1950s, “The Seven Countries Study” (64) has demonstrated MD’s efficacy in MS treatment. A recent meta-analysis reported beneficial effects on: body weight, body mass index (BMI), waist circumference, systolic blood pressure (SBP), diastolic blood pressure (DBP), glucose, insulin, homeostatic model assessment of insulin resistance (HOMA-IR) index, TC, LDL, HDL, triglycerides, alanine transaminase, hepatic fat mass, CRP, IL-6, TNF-α, and flow-mediated dilatation. These determined a lower risk of CVD (RR 0.61, 95% CI: 0.42–0.80) and stroke (RR 0.67, 95% CI: 0.35–0.98) (65).

The idea that many of these benefits may be linked not only to a balanced diet but to the added value of EVOO has prompted many further investigations of EVOO.

Table 1 summarizes the results regarding EVOO’s effects on MS from the main human studies.

**TABLE 1:** Main human studies about EVOO effects on MS, CVD risk, and NAFLD.

Intervention and compounds used	Study design	Dose	Population	Health effect	References
Low fat diet vs. MD + EVOO vs. MD + nuts.	Randomized, controlled, multicenter intervention trial.	EVOO: free, maximum 1 L/week. Nuts: 30 g/day.	7,216 men and women at high cardiovascular risk, aged 55–80.	Subjects in the highest energy-adjusted tertile of baseline total OO and EVOO consumption had 35 and 39% CVD risk reduction, respectively, compared to the reference. Higher baseline total OO consumption was associated with 48% reduced risk of CVD mortality. For each 10 g/d increase in EVOO consumption, CVD and mortality risk decreased by 10 and 7%, respectively.	(9)
EVOO with high polyphenol content (629 mg/L) vs. ROO with null polyphenol (0 mg/L) content.	Randomized, double-blind, crossover trial.	EVOO or ROO: 25 ml/day for 3 weeks.	36 non-smoking males aged 20–60.	Ingestion of EVOO significantly reduced LDL and plasma oxidative markers.	(31)
OO with low (2.7 mg/kg), medium (164 mg/kg), or high (366 mg/kg) phenolic content.	Randomized, crossover, controlled trial.	Three sequences of daily administration of 25 mL of the 3 OOs for 3 weeks.	200 healthy male volunteers aged 20–60.	Linear increase in HDL levels and linear decrease of total cholesterol-HDL cholesterol ratio and oxidative stress markers was observed for low-, medium-, and high-polyphenol OO. Triglyceride levels decreased by an average of 0.05 mmol/L for all OOs.	(32)
MD with OO vs. prudent diet (carbohydrates, 50–60%; proteins, 15–20%; total fat, <30%).	Randomized, single-blind trial.	OO in intervention group: mean 26.7 g/day. OO in control group: mean 15.9 g/day.	180 patients (99 men and 81 women) with MS. 90 patients in intervention group and 90 in control group.	Significant reduction of body weight, hs-CRP, IL-6, IL-7, IL-18 and insulin resistance in intervention group. Endothelial function score improved in the intervention group. MD might be effective at reducing the prevalence of MS and its associated cardiovascular risk.	(49)
Low fat diet vs. MD + EVOO vs. MD + nuts.	Randomized, controlled, multicenter intervention trial.	EVOO: free, maximum 1 L/week. Nuts: 30 g/day.	3,541 men and women without diabetes, aged 55–80, at high cardiovascular risk.	MD enriched with EVOO but without energy restrictions reduced diabetes risk for persons with high cardiovascular risk. In addition, body weight decreased in 80 new-onset diabetes patients assigned to MD plus EVOO intervention group.	(67)
Low fat diet vs. MD + EVOO vs. MD + nuts.	Randomized, controlled, multicenter intervention trial.	EVOO: free, maximum 1 L/week. Nuts: 30 g/day.	191 subjects aged 55–80 at high cardiovascular risk (67 MD + EVOO; 74 MD + nuts; 50 low fat diet).	MD + EVOO and MD + nuts increased adiponectin/leptin ratio values, adiponectin/HOMA-IR ratio, and reduced waist circumference and body weight compared to baseline.	(68)
MD with different quantities of EVOO.	Observational study.	EVOO: from non-consumers to high-consumers.	521,448 healthy volunteers aged between 25 and 70.	MD reduced weight gain.	(69)
Low fat diet vs. MD + EVOO vs. MD + nuts.	Randomized, controlled, multicenter intervention trial.	EVOO: free, maximum 1 L/week. Nuts: 30 g/day.	210 subjects aged 55–80 at high cardiovascular risk (71 MD + EVOO; 68 MD + nuts; 71 low fat diet).	After 1 year, MD + EVOO increased LDL resistance against oxidation, LDL particle size and LDL cholesterol content, reducing the degree of LDL oxidative modifications compared to low-fat control diet. No proven effects for MD + nuts.	(72)
Low fat diet vs. MD + EVOO vs. MD + nuts.	Randomized, controlled, multicenter intervention trial.	EVOO: free, maximum 1 L/week. Nuts: 30 g/day.	418 subjects aged 55–80 at high cardiovascular risk (139 MD + EVOO; 145 MD + nuts; 134 low fat diet).	After median follow-up of 4.0 years, MD + EVOO and nuts groups reduced diabetes incidence compared to control group.	(75)
Two isoenergetic meals with similar composition including EVOO or not.	Randomized, crossover, controlled trial.	EVOO: 10 g. Meal composition: ∼700 kcal; proteins 16–19%, carbohydrates 53–54% and lipids 28–30%.	30 patients (17 males and 13 females, mean age 58.1 ± 11.4 years) with IFG.	EVOO meal was associated with reduction of glucose, triglycerides, Apo B-48 and DPP4 activity and increase of insulin and GLP-1 compared to the meal without EVOO. Total and HDL cholesterol levels did not significantly change between the two groups.	(76)
Low fat diet vs. MD + EVOO vs. MD + nuts.	Randomized, controlled, multicenter intervention trial.	EVOO: free, maximum 1 L/week. Nuts: 30 g/day.	210 subjects aged 55–80 with MS (71 MD + EVOO; 68 MD + nuts; 71 low fat diet).	MD reduced oxidative damage to lipids and DNA in MS individuals. After 1-year urinary F2-isoprostanes decreased in all groups, the decrease in both MD groups reaching a borderline significance vs. low fat diet group. Urinary 8-oxo-7,8-dihydro-2′-deoxyguanosine reduced in all groups, with a higher decrease in both MD groups vs. low fat diet group.	(79)
Analysis of population divided by different quantities of daily OO intake.	Multi-centric European prospective cohort study.	EVOO quartile 1: <10 g/day. EVOO quartile 2: ≥10–<20 g/day. EVOO quartile 3: ≥20.1–<28.9 g/day. EVOO quartile 4: ≥28.9 g/day.	40,142 participants (38% male), free of coronary heart disease events at baseline.	OO intake was negatively associated with coronary heart disease risk for each 10 g/day OO intake, with a more pronounced effect in EVOO consumers.	(80)
High polyphenol EVOO vs. low polyphenol OO.	Randomized, controlled, double-blind cross-over trial.	EVOO or OO: 60 mL/day over two 3-week intervention periods, in conjunction with their habitual diet.	50 healthy subjects aged 38.5 ± 13.9 (66% female).	No significant differences between treatments in total antioxidant and anti-inflammatory effect. However, when the population was stratified by CVD risk status, high polyphenol EVOO showed anti-inflammatory and antioxidative effects compared to low polyphenol OO.	(82)
MD including EVOO vs. habitual diet.	Randomized, controlled, intervention trial.	EVOO: over 14.8 mL/day.	166 men and women aged >64 (85 MD vs. 81 habitual diet).	MD resulted in lower systolic blood pressure at 3 and 6 months compared to habitual diet. FMD was higher by 1.3% in MD group compared to habitual diet.	(83)
Low fat diet vs. MD + EVOO vs. MD + nuts.	Randomized, controlled, multicenter intervention trial.	EVOO: free, maximum 1 L/week. Nuts: 30 g/day.	90 non-smoking women with moderate hypertension aged 60–80 (30 MD + EVOO; 30 MD + nuts; 30 low fat diet).	Diastolic blood pressure reduced with both MD + EVOO and MD + nuts diets. Negative correlation observed between changes in NO metabolite concentration and systolic or diastolic blood pressure in MD + EVOO group. Systolic blood pressure reduction inversely related with ET-1 concentrations in MD + nuts group.	(84)
High polyphenol EVOO vs. low polyphenol OO.	Randomized, controlled, double-blind, cross-over trial.	EVOO or OO: 60 mL/day over two 3-week intervention periods, in conjunction with their habitual diet.	50 healthy subjects aged 38.5 ± 13.9 (66% female).	Significant decrease in peripheral and central systolic blood pressure by 2.5 and 2.7 mmHg, respectively, was observed after high polyphenol EVOO intake. Diastolic blood pressure and arterial stiffness were not influenced by either EVOO or OO intake.	(85)
High polyphenol EVOO vs. low polyphenol OO.	Randomized, controlled, double-blind, crossover trial.	EVOO or OO: 50 ml in single dose as a smoothie consisting of 1/2 cup frozen blueberries and 1 cup low-fat vanilla yogurt.	20 subjects (mean age 56.1; 10 women, 10 men) at risk for diabetes (either prediabetes or MS).	FMD as marker of endothelial function was measured 2 h after the meal; EVOO acutely improved FMD as compared to OO. No significant effects on systolic or diastolic blood pressure were observed.	(86)
Low glycemic index MD vs. control diet.	Randomized, controlled, double-blind, clinical trial.	OO: no specific quantity reported.	98 subjects with moderate or severe NAFLD (50 low glycemic index MD and 48 control diet).	Negative interaction between time and low glycemic index MD was observed on the NAFLD score, becoming more evident at the sixth month.	(91)
MD with OO and nuts vs. low fat diet.	Randomized single-blind, controlled trial.	OO: approximatively 25 mL/day (750 ml provided every month). Nuts: approximately 25 g/day (750 g provided every month).	49 subjects with NAFLD, mean age 52 (26 MD group, 25 low fat diet).	After 12 weeks hepatic steatosis reduced significantly in both groups and no difference in liver fat reduction between groups, with mean relative reductions of 25.0% in low fat diet and 32.4% in MD. Liver enzymes also improved significantly in both groups.	(92)
MD with OO vs. low fat diet.	Randomized, controlled, crossover, trial.	OO: approximatively 16.6 mL/day (500 ml provided every month).	Twelve non-diabetic subjects (50% female) with biopsy-proven NAFLD.	After 6 weeks there was a significant relative reduction in hepatic steatosis with MD compared to low-fat diet. Insulin sensitivity improved with MD, whereas no change was proven after low fat diet.	(93)
MD + EVOO with high oleocanthal.	Single center, prospective cohort study.	EVOO with high oleocanthal: 32 g/day.	23 subjects with the MS and hepatic steatosis (15 men and 8 women, age: 60 ± 11).	After 2 months there was a reduction of body weight, waist circumference, alanine transaminase, IL-6, IL-17A, tumor necrosis factor-α, and IL-1β, while IL-10 increased. Maximum subcutaneous fat thickness increased, with a concomitant decrease in the ratio of visceral fat layer thickness/subcutaneous fat thickness max.	(94)
Low fat diet vs. MD + EVOO vs. MD + nuts.	Randomized, controlled, multicenter intervention trial.	EVOO: free, maximum 1 L/week. Nuts: 30 g/day.	100 subjects aged 55–80 at high cardiovascular risk (34 MD + EVOO; 36 MD + nuts; 30 low fat diet).	After 3 years of intervention, MD + EVOO group showed significantly lower hepatic steatosis compared to other groups even though mean values of liver fat content were not statistically different.	(95)

CRP, C-reactive protein; CVD, cardiovascular disease; DPP4, dipeptidyl-peptidase-4; ET, endothelin; EVOO, extra virgin olive oil; FMD, flow mediated dilatation; GLP-1, glucagon-like peptide-1; HDL, high-density lipoprotein; HT, hydroxytyrosol; IFG, impaired fasting glucose; IL, interleukin; MD, mediterranean diet; NAFLD, non-alcoholic fatty liver disease; NO, nitric oxide; OO, olive oil; ROO, refined olive oil.

### Effects on body weight and adipose tissue

Mediterranean diet contributes to body weight reduction with a consequent decrease in BMI and visceral obesity (49), but there is insufficient evidence to indicate whether EVOO, by itself, influences these parameters (66). In the PREDIMED study, body weight and waist circumference decreased in patients assigned to MD + EVOO intervention group (67, 68).

The EPIC-PANACEA study found that following an MD including different quantities of EVOO reduced weight gain (69). EVOO may assist in body weight reduction because of its organoleptic qualities which enhance food palatability and promotes satiety (70).

*In vitro* HT supplementation of cultures of Simpson–Golabi–Behmel syndrome human pre-adipocytes, modulated gene expression, reducing NF-κB and oxidative stress pathway activation, decreasing inflammation and macrophage recruitment (71).

Therefore, EVOO seems to act more as a counter-regulator of adipose tissue inflammation than as a reducer of visceral obesity.

### Effects on lipid metabolism

Extra virgin olive oil can modify lipidic metabolism, optimizing circulating cholesterol and triglyceride levels (6, 39) and reducing LDL oxidation (31, 32).

In a randomized trial, MD associated with 8 g/day OO intake for 2 years reduced triglycerides (*p* = 0.001) and TC (*p* = 0.02) levels and increased HDL (*p* = 0.03) (49). It was demonstrated that 1 year of MD + EVOO intake enhanced LDL resistance to oxidation (*p* = 0.007), reduced changes related to oxidative stress (*p* < 0.05), increased their size (*p* = 0.021) and cholesterol content (*p* = 0.013) compared to a low-fat diet (72). Researchers comparing 200 healthy volunteers divided into three groups, each with an intake of progressively higher polyphenol content OO, demonstrated that HDL levels increased linearly with the concentration of polyphenols; similarly, TC, TC/HDL ratio, and LDL oxidation decreased (32).

Recently a network meta-analysis of OO metabolic effects, as part of MD, evaluated 30 human intervention studies, considering direct and indirect interactions and impact of OO constituents over different metabolic pathways. Effects on glucose, triglycerides, and LDL-C were mediated by adherence to MD, whereas polyphenols increased HDL-C and improved antioxidant and inflammatory status as an independent factor. Interestingly, benefits were more pronounced in subjects with MS or chronic conditions/diseases than healthy subjects (73).

Briefly, we can assume that MD produces a protective effect against lipid-induced atherogenesis by reducing LDL-C, while the added value of EVOO mainly increased HDL-C and prevents LDL oxidation. However, more extensive studies with well-defined EVOO quantities and chemical characteristics (27), are needed in order to confirm these findings.

### Effects on glycidic metabolism

A meta-analysis of four cohorts and 29 trials associating EVOO with glycemic control found the risk of diabetes mellitus (DM) was inversely associated with EVOO intake (*p* < 0.01), though non-linearly (*p* < 0.01) (74). The risk of developing DM decreased by 13% with EVOO intake of 15–20 g/day, but no advantages from further dose increases were proven. EVOO trial analysis showed significantly more evident reductions in HbA1c (*p* < 0.01) and fasting blood glucose (*p* < 0.01) than control groups.

The PREDIMED study confirmed that MD + EVOO can improve glucose metabolism, preventing diabetes onset (75). In another subgroup of patients from PREDIMED study MD + EVOO and MD + nuts increased values of adiponectin/leptin ratio (*p* = 0.001 and *p* < 0.001, respectively) and adiponectin/HOMA-IR ratio (*p* = 0.027 and *p* = 0.069, respectively) compared to baseline (68).

Extra virgin olive oil should influence glucose metabolism, reducing dipeptidyl peptidase-4 activity and resulting in an increase in the glucagon-like peptide-1 incretin-pattern (76). Moreover, EVOO’s polyphenols might partially inhibit carbohydrate digestion and absorption, reducing the hepatic release of glucose and increasing its peripheral uptake (77). Polyphenol antioxidant activity might reduce the production of advanced glycosylated end-products such as HbA1c (78).

Taken together, both MD and EVOO seem to optimize glucose metabolism, ensuring better glycemic control and reducing DM risk. However, EVOO seems to provide additional protection, specifically acting on insulin secretion mechanisms. Nevertheless, most investigations of specific polyphenol mechanisms in regulating glycidic metabolism were conducted in *in vitro* models.

### Effects on the cardiovascular system

Many studies investigated the effects of MD and EVOO on cardiovascular risk (49, 79). In the PREDIMED study, patients with the highest EVOO intake showed a 39% reduction in CVD risk. Risk of CVD and CVD death was also reduced by 10 and 7%, respectively, for each 10 g/day increase in EVOO intake (9).

The EPIC cohort study also provides relevant data. Buckland et al. (80) analyzed data from 40,142 coronary disease-event free participants, showing that OO intake during follow-up was negatively associated with coronary disease risk for each 10 g/day OO intake, with a more pronounced effect from EVOO (14% risk reduction, *p* = 0.072).

More recently, a double-blind randomized, controlled, cross-over study (OLIVAUS) evaluated the effects of high polyphenol EVOO vs. low polyphenol OO on CVD in 50 healthy participants (81). When the population was stratified by CVD risk status, EVOO showed anti-inflammatory and antioxidative effects only during high polyphenol EVOO intake (*p* = 0.0086). In detail, the subgroup with abdominal obesity showed reduced oxidated LDL and increased total antioxidant capacity (82).

Some authors reported a positive effect of MD + EVOO on blood pressure control; a reduction in SBP values both at 3 and 6 months was reported (83), as well as a negative correlation between changes in NO metabolite concentration and SBP-DBP pressure values compared to baseline in the MD + EVOO group (*p* = 0.033 and *p* = 0.044, respectively) (84). The OLIVAUS study showed reduced SBP during high polyphenol EVOO intake rather than low polyphenol OO. These effects disappeared after the challenge ended, connecting blood pressure reduction to continuous EVOO intake (85).

In this context, a randomized, controlled, double-blind, crossover trial on 20 adults with MS evaluated the effect of high polyphenol EVOO vs. a refined oil without polyphenols on endothelial function. Flow-mediated dilatation measurement was used to analyze endothelial function after a single 50 ml dose of one of the 2 OOs. EVOO improved endothelial function (*p* = 0.0086) compared to refined oil, though it had no significant effects on SBP-DBP; this last data may be related to the single dose and should be analyzed in long-term studies (86).

Although studies report a consistent reduction of CVD risk and mortality, probably related to both MD and EVOO intake, the high variability of EVOO effects on SBP-DBP and its regulation mechanisms (NO and endothelin-1) requires further and larger human studies. Available data do not clarify whether blood pressure modification is acute or chronic, or if there is a threshold effect or tachyphylaxis, or long-term tolerance.

### Effects on liver steatosis

Non-alcoholic fatty liver disease (NAFLD) and non-alcoholic steatohepatitis (NASH) represent the hepatic manifestation of MS (87). The link between these two conditions is so high that several researchers prefer the term metabolic associated fatty liver disease (MAFLD) (88).

A 5–7% of body weight decrease can reduce liver steatosis (89). Thus, a balanced MD would seem to have a certain impact on NAFLD/MAFLD. A recent meta-analysis of 18 studies, considering three different dietary patters (western diet, prudent diet, and MD) found an increased risk of NAFLD by 56% for western diet and a reduced risk of this disease by 22 and 23%, respectively in prudent diet and MD (90).

Some studies considered an MD-based dietary intervention compared to a low-fat low-carbohydrate diet in patients with NAFLD (91–93). A recent meta-analysis compared these studies, proving a consistent reduction of the intrahepatic lipid content in intervention groups (mean difference: −0.57, 95% CI: −1.04 to −0.10) (89). However, no difference between groups were proved in alanine aminotransferase and γ-glutamyl transpeptidase level reduction.

Patti et al. (94) analyzed the effectiveness of a 2-month intervention with EVOO (32 g/day) in subjects with MS and associated liver steatosis, showing a reduction of alanine aminotransferase levels (*p* = 0.029) after intervention, and considered it a possible indirect demonstration of liver steatosis reduction. The PREDIMED cohort randomized patients in three subgroups: MD + EVOO, MD + nuts, and control diet. After 3 years, liver steatosis was present in 8.8, 33.3, and 33.3% of the subgroups (*p* = 0.027), and mean liver fat content values were 1.2, 2.7, and 4.1% (*p* = 0.07), respectively (95).

These data indicate that MD has beneficial effects on NAFLD/MAFLD, whereas there is poor, contrasting, and mainly indirect evidence that EVOO could effectively influence liver fat content. Thus, larger randomized studies with specific EVOO quantities and composition are required to clarify its role in MAFLD.

## Conclusion

Mediterranean diet is a cornerstone in treating MS and preventing cardiovascular risk. Literature data indicate that an essential component is EVOO which, with high MUFA and polyphenol content constitutes a food with excellent organoleptic properties and a substance with surprising nutraceutical abilities. EVOO, by activating multiple metabolic pathways, could optimize glycemic control and lipid metabolism, reduce endothelial damage and blood pressure, and provide systemic anti-inflammatory activity.

Overall, EVOO seems to play an antiatherogenic and CVD risk reduction role, improving the overall health status of MS patients. Given its ability to modulate inflammatory stress, some studies are evaluating EVOO activity in cancer (e.g., breast cancer) (96).

As of June 2022, 32 trials on EVOO’s effects in several pathological conditions have been registered on ClinicalTrials.gov,^1^ of which 11 are in the active recruitment phase; the conditions investigated include: CVDs, MS, end-stage renal failure, autoimmune diseases, breast cancer and mitochondrial diseases.

Though the evidence supporting a role of EVOO and its polyphenolic component in MS is increasing rapidly, a recent meta-analysis of 76 trials, found no significant effect of OO, HT, and oleic acid on MS, considered both overall and in its different components. Statistical significance was only shown for OO, HT, and oleic acid’s antioxidant capacity related to components of MS. However, most studies compared OO with other MS treatment approaches, so the lack of statistical significance indicates OO’s non-inferiority rather than non-efficacy (97).

Several doubts remain regarding EVOO’s action mechanisms, the quantities required to optimize its effects and, whether its properties can be separated from those of MD or if the beneficial effects are inextricably linked.

Tsartsou et al.’s (73) meta-analysis provides two compelling indications which need confirmation by prospective studies. First OO’s effects on glucose, triglycerides, and LDL-C were mediated by adherence to MD, whereas polyphenol effects seem to be limited to increasing HDL and modulating oxidative stress and inflammation. Second, polyphenol effects do not seem to be directly correlated to their levels in OO, such that a much lower than previously reported concentration of OO polyphenols can induce protection.

This last result conflicts with recent nutrigenomic studies showing that EVOO cultivars with high polyphenol content can modulate the expression of several transcripts involved in glucose/lipid metabolism, proliferation, inflammation, and cancer, supporting health-promoting effects pathways (70).

We must stress that most human studies of EVOO activity inadequately characterize biochemical features of the EVOO used, especially different phenolic concentrations which consistently differ across varieties (27, 70). This point, together with the differences in daily EVOO intake (which in some studies is not standardized) could influence the results of human clinical trials.

Another shadowy point regards the bioavailability of EVOO compounds (98). Polyphenol absorption in the gut seems to be dose- and time-dependent and is strictly dependent on their chemical structure’s polarity (18–20, 99). Furthermore, the specific individual characteristics of intestinal microbiota can influence the bioavailability of phenolic compounds (30, 100–102). Finally, studies reported that olive cultivars might modify the bio-accessibility and antioxidant activity of EVOO’s phenolic fraction (101, 102).

Thus, considering all these issues, larger, well-structured and standardized (e.g., EVOO quantities and chemical features) studies are required to clarify EVOO’s potential as a nutraceutical product.

## Author contributions

AS, MS, and MC: full access to all the data in the study, take responsibility for the integrity of the data, the accuracy of the data analysis, and methodology. AS, VD, and MC: conceptualization. AS, LM, ACu, and GA: investigation and literature research. AS and VD: writing – original draft preparation. AS, MS, LG, VD, RC, ACu, GA, ACa, JI, and MC: writing – review and editing. LG and MS: funding acquisition. All authors contributed to the article and approved the submitted version.

## Footnotes

1
https://www.clinicaltrials.gov/ct2/results?cond=&term=evoo&cntry=&state=&city=&dist=

## Abbreviations

ATP III: Adult Treatment Panel III
COX: cyclooxygenase
CRP: C-reactive protein
CV: cardiovascular
CVD: cardiovascular disease
DBP: diastolic blood pressure
DM: diabetes mellitus
EFSA: European Food Safety Authority
EVOO: extra virgin olive oil
HDL: high-density lipoproteins
HT: hydroxytyrosol
IFN: interferon
IL: interleukin
LDL: low-density lipoproteins
LDL-C: low-density lipoproteins-cholesterol
MAFLD: metabolic associated fatty liver disease
MD: mediterranean diet
MS: metabolic syndrome
MUFA: monounsaturated fatty acids
NAFLD: non-alcoholic fatty liver disease
NASH: non-alcoholic steatohepatitis
NCEP: National Cholesterol Education Program
OO: olive oil
PUFA: polyunsaturated fatty acids
SBP: systolic blood pressure
TC: total cholesterol
TNF: tumor necrosis factor
Tyr: tyrosol.
