# Supplementary material for: The clinical impact of an extra virgin olive oil enriched mediterranean diet on metabolic syndrome: Lights and shadows of a nutraceutical approach
Source: Front Nutr. 2022 Aug 4;9:980429. doi: 10.3389/fnut.2022.980429 (PMC9386289; doi:10.3389/fnut.2022.980429)
Supplement: Supplementary file 1 [file Table_1.DOCX]

**Supplementary Table 1**: Phenolic compound subclasses

| **Subclasses** | **Molecules** |
| --- | --- |
| **Phenolic acids** | Caffeic Acid  Ferulic Acid  Gallic acid  Gentisic acid  o-Coumaric acid  p- Hydroxybenzoic acid  p-Coumaric acid  Syringic acid  Vanillic acid |
| **Secoiridoid aglycons** | Decarboxymethyl ligstroside aglycon  (p-HPEA-EDA or oleocanthal)  Decarboxymethyloleuropeinaglycon  (3,4-DHPEA-EDA or oleacein)  Dialdehydic form of ligstroside aglycon  Dialdehydic form of oleuropein aglycon  Ligstroside aglycon (p-HPEA-EA)  Oleuropeinaglycon (3,4-DHPEA-EA) Oleaceinic acid  Oleocanthalic acid  Oleokoronal  Oleomissional |
| **Flavones** | Apigenin  Luteolin  Methyl-luteolin |
| **Lignans** | Acetoxypinoresinol  Pinoresinol |
| **Phenolic alcohols** | Hydroxytyrosol (3,4-DHPEA)  Tyrosol (p-HPEA) |
